# Retroviral Elements in Pathophysiology and as Therapeutic Targets for Amyotrophic Lateral Sclerosis

**DOI:** 10.1007/s13311-022-01233-8

**Published:** 2022-04-12

**Authors:** Wenxue Li, Darshan Pandya, Nicholas Pasternack, Marta Garcia-Montojo, Lisa Henderson, Christine A. Kozak, Avindra Nath

**Affiliations:** 1 Section of Infections of the Nervous System, National Institute of Neurological Disorders and Stroke, Bethesda, MD USA; 2 National Institute of Allergy and Infectious Diseases, National Institutes of Health, Bethesda, MD USA

**Keywords:** Human endogenous retrovirus, Retrotransposons, Transposable elements, Motor neuron disease, Murine leukemia virus, Human immunodeficiency virus, Antiretroviral drugs, Frontotemporal dementia

## Abstract

The study of the role of retroviruses in amyotrophic lateral sclerosis (ALS) dates back to the 1960s shortly after transposable elements themselves were first discovered. It was quickly realized that in wild mice both horizontal and vertical transmissions of retroviral elements were key to the development of an ALS-like syndrome leading to the postulate that endogenous retroviruses (ERVs) contribute significantly to the pathogenicity of this disease. Subsequent studies identified retroviral reverse transcriptase activity in brains of individuals with ALS from Guam. However, except for a single study from the former Soviet Union, ALS could not be transmitted to rhesus macaques. The discovery of an ALS-like syndrome in human immunodeficiency virus (HIV) and human T cell leukemia virus infected individuals led to renewed interest in the field and reverse transcriptase activity was found in the blood and cerebrospinal fluid of individuals with sporadic ALS. However, exogenous retroviruses could not be found in individuals with ALS which further reinforced the possibility of involvement of a human ERV (HERV). The first demonstration of the involvement of a HERV was the discovery of the activation of human endogenous retrovirus-K subtype HML-2 in the brains of individuals with ALS. The envelope protein of HML-2 is neurotoxic and transgenic animals expressing the envelope protein develop an ALS-like syndrome. Activation of HML-2 occurs in the context of generalized transposable element activation and is not specific for ALS. Individuals with HIV-associated ALS show a remarkable response to antiretroviral therapy; however, antiretroviral trials in ALS down-regulate HML-2 without ameliorating the disease. This highlights the need for specific drugs to be developed against HML-2 as a novel therapeutic target for ALS. Other approaches might include antisense oligonucleotides, shRNA targeted against the envelope gene or antibodies that can target the extracellular envelope protein. Future clinical trials in ALS should consider combination therapies to control these ERVs.

**Supplementary Information:**

The online version contains supplementary material available at 10.1007/s13311-022-01233-8.

## Introduction

Amyotrophic lateral sclerosis (ALS) is a neurodegenerative disease that specifically affects upper and lower motor neurons. There are several unique aspects of this illness. There is a clear anatomical spread in a pattern that is consistent with a contiguous spread of the disease process in the motor neuron network resulting in progressive motor weakness [1]. While the motor system including the premotor networks and the frontal cortex are particularly vulnerable, other parts of the brain are relatively spared. The disease progresses rapidly with nearly 90% mortality within 5 years. Creutzfeldt–Jakob disease (CJD) is the only other neurodegenerative disease that progresses more rapidly than ALS. CJD was initially thought to be due to a slow virus but later identified as a prion.

The etiopathogenesis of ALS remains unresolved. The possibility that ALS is caused by an infectious agent has long been considered, and, despite evidence suggesting that possibility, all attempts to isolate the infectious agent have been to no avail. However, reverse transcriptase activity has been found consistently in these individuals even before the term, “reverse transcriptase” was coined. This retrovirus encoded enzyme is required for virus replication and it has long been speculated that the source of the enzymatic activity might be an endogenous retrovirus (ERV). In this review, we discuss the complexity of the ERVs in the human genome, provide a historical perspective on the discovery of retroviral expression in ALS and discuss other retroviruses that can cause an ALS-like syndrome. We also provide rationale for targeting retroviral elements as a therapeutic approach to ALS and propose methods by which this can be achieved.

## Transposable Elements and Endogenous Retroviruses

**Fig. 1:**
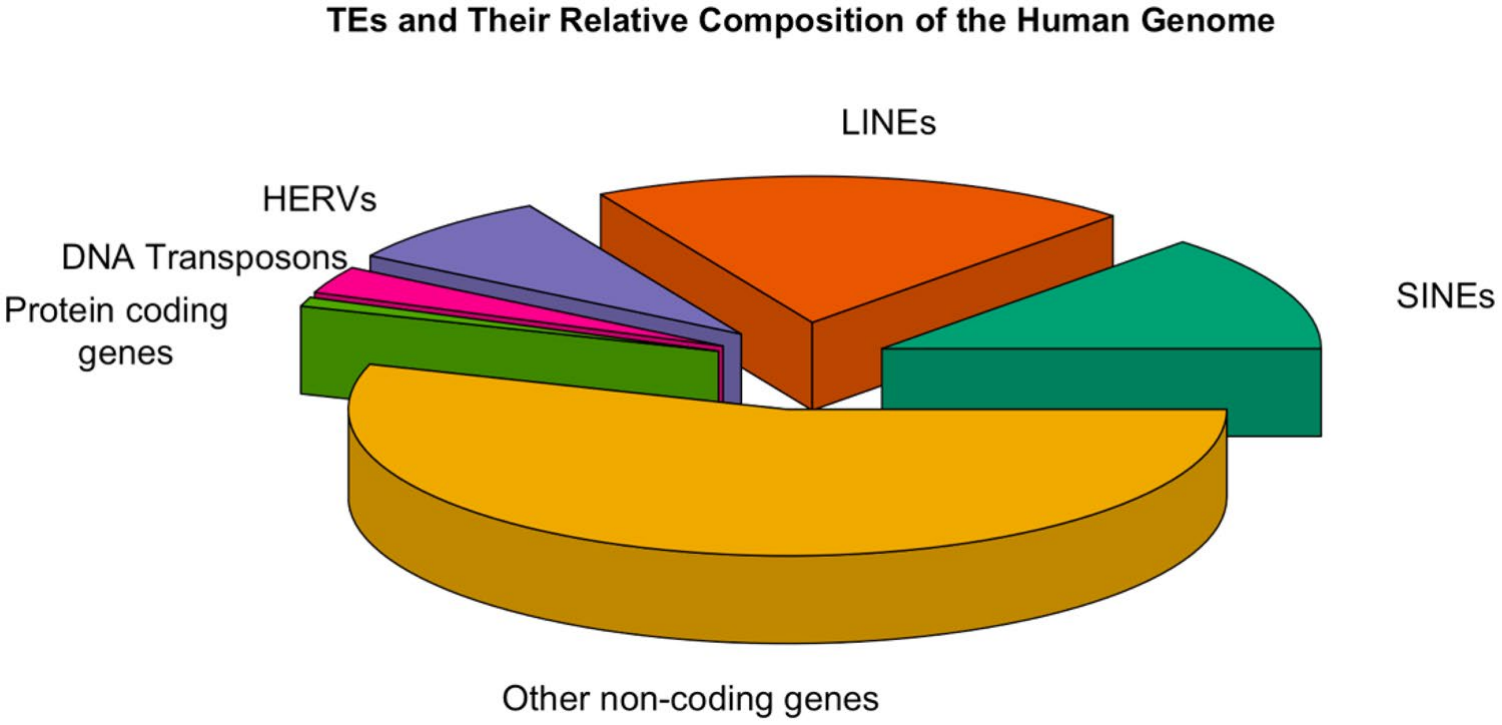
Transposable elements and their relative composition of the human genome: Relative composition of genes of the human genome based on values from [3]. The DNA transposons; LTR retrotransposons (HERVs); protein coding genes; and the non-LTR retrotransposons long interspersed nuclear elements (LINEs); and short interspersed nuclear elements (SINEs) are shown

**Fig. 2:**
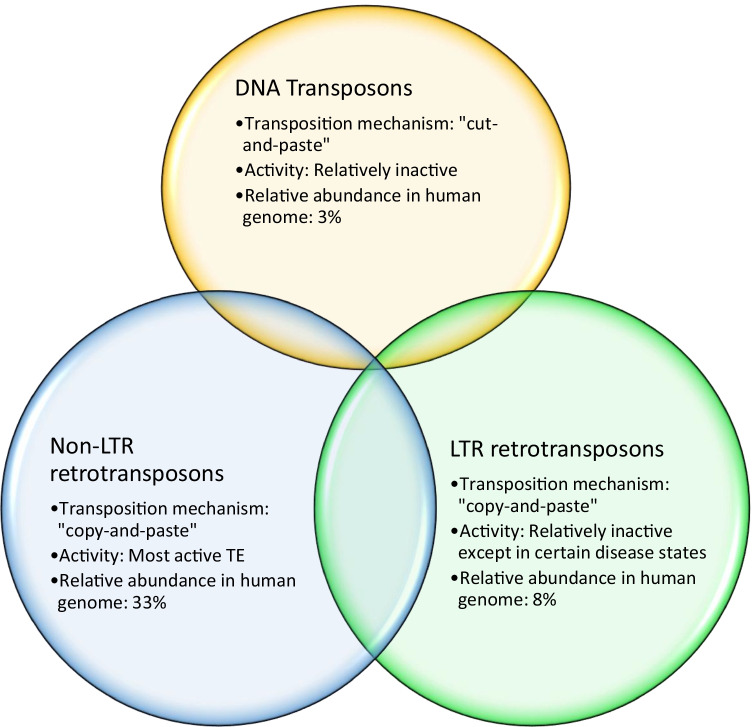
Properties of transposable elements: A Venn diagram of the three main types of TEs discussed in this review: DNA transposons and LTR and non-LTR retrotransposons. The degree of overlap of circles within the Venn diagram indicates relatedness of the TE

**Fig. 3:**
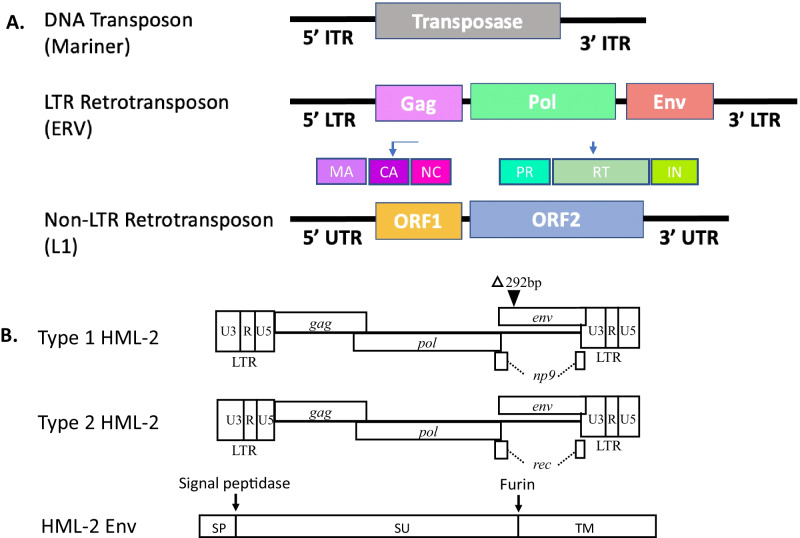
General structure of Transposable elements: (**A**) DNA transposons (top), LTR retrotransposons (middle) and non-LTR retrotransposons (bottom) based on [125]. DNA transposons use the transposase enzyme for “cut-and-paste” transposition and are flanked by inverted terminal repeats (ITRs). LTR retrotransposons encode reverse transcriptase and integrase enzymes which are required for “copy-and-paste” transposition in the Pol domain as well as structural (group specific antigen, Gag) and in many cases envelope (Env) proteins. Non-LTR retrotransposons have open reading frames (ORFs), including ORF2 which encodes reverse transcriptase and endonuclease for transposition and are flanked by untranslated regions (UTRs). (**B**) Type 1 HML-2 has a 292 base pair deletion in the *env* and encodes for *np9*. Type 2 HML-2 has a full length *env* and encodes for *rec*

In contrast to the prevailing perspective of the early twentieth century that viewed genetic material as fixed, in the 1940s Nobel laureate Barbara McClintock discovered the “mutable loci” *Ds* (Dissociation) and *Ac* (Activator) in maize that were capable of moving between chromosomes [2]. McClintock’s pioneering study paved the way for future research into the role of these “jumping genes” or transposable elements (TEs) in both health and disease states. In fact, it was later discovered that 45% of the human genome is comprised of TEs [3] (Fig. 1). There are two main classes of TEs: Class I TE are called retrotransposons and they comprise about 40% of the human genome. Class II TEs are called DNA transposons and they comprise about 3% of the human genome [3]. DNA transposons, although believed to be currently inactive in humans, have the potential to extract themselves as DNA from one locus and then reinsert at another locus via a “cut-and-paste” mechanism [4]. In contrast to the DNA transposons, retrotransposons use a “copy-and-paste” mechanism whereby an RNA intermediate is used to transfer the original DNA sequence to another genetic locus [5] (Fig. 2). Retrotransposons can themselves be divided into two groups: those with a repetitive sequence at either end, the long terminal repeat (LTR) retrotransposons, and those that do not, the non-LTR retrotransposons. Non-LTR retrotransposons include the retrovirus-like long interspersed nuclear elements (LINEs) that are the only family of retrotransposons that can transpose autonomously [6]; and the short interspersed nuclear elements (SINEs), which are primate-specific genes that require protein machinery from long interspersed nuclear element (LINE)-1 (L1) in order to replicate and includes the Arthrobacter luteus (*Alu*) element which has a copy number in the human genome of over 1 million [7]. Non-LTR retrotransposons of interest are L1, *Alu*, and SVA (named due to its primary components: SINE, Variable number tandem repeats and *Alu*) as they are thought to be the most active type of TEs in humans [8]. In fact, only about 0.05% of retrotransposons are thought to be active in humans and these are believed to include *Alu*, L1 and SVA [9] (Fig. 3).

LTR retrotransposons include the ERVs and are found in all vertebrates including humans where they are known as human endogenous retroviruses (HERVs). In fact, up to 10% of the vertebrate genome consists of ERVs [10], and HERVs comprise about 8% of the human genome [3] (Fig. 1). The LTR portion of LTR retrotransposons can also exist as “solo LTRs” [11]. Solo LTRs can be generated through a homologous recombination between the two LTRs flanking the provirus and subsequent deletion of the internal sequence [12].

How did HERVs find their way into the human genome and why are they so prevalent? At different points in human evolutionary history, exogenous retroviruses infected our primate ancestors, occasionally infecting a germline cell which allowed for vertical transmission of the element from parent to offspring [13]. As the HERVs have been constituents of our DNA for millions of years, they have accumulated numerous mutations and undergone homologous recombination events to create mosaic forms. As a result, most HERV elements have lost their coding capacity and are incapable of making protein as they lack open reading frames [14]. Although complete, infectious HERV viral particles have not been identified in humans, reactivation of HERVs and formation of viral proteins have been associated with several diseases. However, gamma-retrovirus ERVs in mice can produce viruses that can similarly cause disease [15, 16]. A HERV subgroup of particular interest in the field of neurodegenerative diseases is HERV-K (named for its lysine, abbreviated K, tRNA primer). HERV-K is a member of the beta-retrovirus-like HERV and has 11 subtypes called HML-1 through HML-11 [17].

**Fig. 4:**
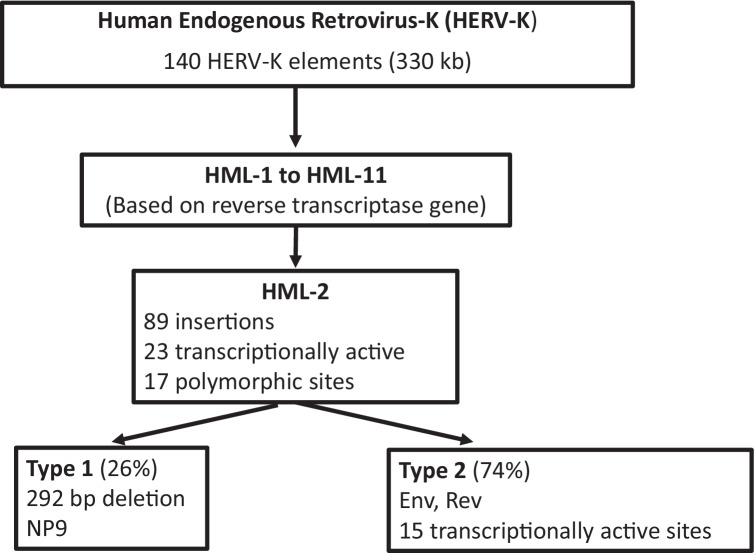
Classification of Human Endogenous Retrovirus-K (HERV-K): HERV-K has been classified into 11 subgroups based on sequence of the reverse transcriptase. HML-2 is the most recent insertion and the most intact retrovirus in the human genome. It has multiple insertions and based on the sequence of the envelope gene is further classified into two subtypes

HERV-K (HML-2) is classified as a beta-retrovirus and is one of the newer HERV additions to our genome. HML-2 has been shown to contain coding viral genes as well as a full-length virus with the potential to replicate in humans [18]. HML-2 can further be classified into type 1 and type 2 depending on the presence or absence of a 292-bp deletion that spans the *pol–env* junction. Type 2 has a full length or nearly full length envelope sequence and encodes the Rec protein. Type 1, with the 292-bp deletion and is unable to produce the envelope or Rec but instead produces NP9 (Fig. 3B). There are also some HML-2 proviruses with larger deletions. There are at least 89 HML-2 proviruses, of which 26% are type 1 and 74% are type 2. There are 23 transcriptionally active HML-2 proviruses in the human genome (Fig. 4). There are another 36 non-referenced insertions with frequencies ranging from < 0.0005 to 0.75 in the human genome. 17 HML-2 sites are polymorphic insertions in humans. There are 15 proviral insertions named for their chromosomal location, that have an open reading frame for the envelope (env) sequence and are transcriptionally active[17]. Of these, the *env* at 22q11.23 is a full length sequence and is expressed in teratocarcinoma [19]; the 19q11 *env* has no deletion or premature stop codon and is expressed in pleuripotent stem cells [20] and atypical teratoid rhabdoid tumors [21]; 12q14.1 has a full length transcript except for a 3 nucleotide in frame deletion and is also expressed in stem cells although at a lower level [20]; 7p22.1a encodes a full length functional envelope protein and has been implicated in ALS [22]. Therefore, when studying the expression of HERV-K or HML-2 in any disease state including ALS it is critically important to identify all the active loci. Variability in expression at each loci and sites of proviral insertions may vary between individuals. A limitation of Hi seq or high depth sequencing is that the length of the sequences is often too short to be able to match or assign them to a single locus hence meaningful comparisons cannot be made between patients and controls. For this reason, it is important to clone and sequence the transcripts as has been done in some studies [22, 23] but not in others [24, 25]. Other alternatives to be explored include technologies for sequencing long transcripts, such as PacBio (Pacific Biosciences, USA) and Nanopore (Oxford Nanopore Technologies, UK).

TEs cause a variety of diseases in humans including Hemophilia type A by disrupting normal gene function; and cancers and developmental diseases by causing genomic rearrangements [26]. Despite these associations, attempts to measure TE expression levels in healthy control and disease tissue samples using high-throughput approaches present technical challenges. This is principally due to the relatively small amount of transcript copies from any given TE locus and the high variability of TE expression in healthy people. Fortunately, recent advancements in computer-based analysis methods have improved our ability to measure these features in bulk or single cell RNA sequencing and genomic data [27–29]. Using these new methods in conjunction with machine learning approaches has provided and will likely continue to provide additional insight into the role of TEs in disease. Most notably, it was recently shown that 20% of individuals with ALS have TE dysregulation in the motor cortex as a primary feature of their disease [30].

## History of Detection of Retroviral Elements in ALS:

### Transmission of ALS to Non-Human Primates:

The possibility of an infectious particle as the causative agent in the etiology of ALS was first explored in the 1950s. Multiple failed attempts were made to transfer ALS to mice and guinea pigs by intracerebral inoculation of brain homogenates from brain and spinal cord of individuals who had died of ALS. Attempts to isolate the presumably infectious agent by blind passage of brain tissue in mice, chick-embryos, HeLa cells and monkey kidney cells also failed. However, in 1956, Zil’ber and colleagues in Moscow initiated a series of experiments designed to transmit ALS to rhesus macaques. They injected homogenates from the medulla and spinal cord of a patient who had died of bulbar ALS into the frontal cortex of one to two year old rhesus macaques. Two and a half years after injection, one of the animals developed progressive weakness with atrophy of the muscles and brisk reflexes suggestive of both upper and lower motor neuron involvement which is suggestive of an ALS like illness. The weakness spread from the left hind limb to the left forelimb and then the right side over a few months. This type of anatomical spread is commonly seen in ALS. Three years after the initial injection the animal was killed and spinal cord and brainstem homogenates from this animal were injected into four other macaques. Two of the animals developed pathology consistent with motor neuron disease: one at eight months and another at three years post-injection. The infection could then be passed on to another animal by intracerebral inoculation that developed progressive upper and lower motor neuron signs. Histology of the spinal cord showed lack of staining of the lateral corticospinal tract and inclusion bodies in the anterior horn cells which are typical pathological findings in ALS. Multiple other attempts were made to transmit homogenates from ALS brain and spinal cord to several other species of animals, but none were successful. They concluded that a viral agent may be pathogenic in the disease but host genetics played a key role in the manifestation of the illness [31]. These observations caught the attention of United States Public Health Service that sent a delegation of researchers consisting of Jacob Brody, William Hadlow, John Hotchin, Richard Johnson, Hiliary Koprowski and Leonard Kurkland to visit Zil’ber in 1964. They examined three inoculated monkeys that showed brisk reflexes and atrophy but noted that there was no bulbar involvement, and they were unable to confirm the pathological changes reported earlier. They also stated that the “transmissible agent” had been made available to the National Institute of Neurological Diseases and Blindness (now called the National Institute of Neurological Disorders and Stroke at the National Institutes of Health) [32]. Here Gibbs and Gajdusek failed to transmit homogenates of brain and spinal cord from a rhesus macaque who had died of an ALS-like syndrome obtained from Zil’ber into ten other rhesus macaques. They noted that the “specimens had been collected, preserved and processed in a manner similar to that described in detail for Kuru.” In the same report they were unable to transmit Guam ALS using brain homogenates (n = 5) and using blood (n = 4) or brain (n = 2) from individuals with ALS in the USA [33, 34]. While these experiments may have settled the debate in those days as regards the transmissibility of ALS, we now know that brain material from prion mediated diseases such as Kuru and CJD can withstand very harsh conditions and still remain infectious. However, if the transmissible agent in ALS were to be a virus or RNA–protein complex, it would easily degrade unless the material was used fresh or flash frozen and then thawed in RNAse free conditions. Hence the potential transmissibility of ALS remains far from settled.

### Horizontal Human to Human Transmission:

To date there is no epidemiological evidence for the potential transmission of ALS by blood transfusion or organ transplantation. Similarly, while there is no evidence of transmission of CJD by blood transfusion [35], there is evidence of transmission by exposure to brain tissue or dura mater from individuals with the disease [36] or corneal transplants [37]. There is also evidence for transmission of CJD in recipients of human pituitary extracts [38]. Further nearly 50% of recipients of human growth hormone from pituitary extracts or cadaveric dura mater grafts had accumulation of amyloid beta peptide leading to an Alzheimer’s disease-like pathology and cerebral amyloid angiopathy raising the possibility that these diseases may also be transmissible [39–41]. In the US cohort of individuals who had received human growth hormone from pituitary extracts there were three cases of ALS. All cases were relatively young at the time of death (ages 30, 30 and 23 yrs) and the disease was rapidly progressive [42, 43]. These features raise the possibility of human-to-human transmission of ALS, although, the possibility that these cases might have occurred by chance alone cannot be entirely excluded. Careful continued tracking of ALS and frontotemporal dementia cases in the US and European cohorts who received human pituitary extracts or were exposed to diseased human CNS tissues are necessary.

### Retroviruses and ALS like Syndrome in Mice:

One of the earliest detailed reports in the literature is by PG Stansly in 1965 of a “paralytic disease associated with virus-induced neoplasms of the mouse”. In this report, he describes the history of the discovery, the clinical features, the pathology, the long latency and the effect of the age of the host on the development of the syndrome by a filterable agent that was also associated with lymphomas [44]. He showed that the infectious agent could be transmitted to mice at an early age and had a latency of 6–12 months. Balb/c mice are more susceptible compared to some of the other strains and the mice developed paralysis and spasticity without sensory involvement and histopathology showed loss of anterior horn cells. This confirmed the involvement of both upper and lower motor neurons suggestive of an ALS like illness [44]. It is well known that gamma-retroviruses cause lymphoma and leukemias in mice as well as neurological disease and immunodeficiencies, while mouse beta-retroviruses cause breast cancer. It was subsequently found that these pathogenic agents could be transmitted both horizontally and vertically, and that endogenized viruses if reactivated could cause cancer. Murray Gardner and colleagues characterized such viruses from wild mice, and discovered that some populations of mice carrying a naturally occurring murine leukemia virus (MuLV) developed motor paralysis and lymphoma while other developed motor paralysis alone. Viremia occurred in these mice occurred at or shortly after birth but neurological symptoms only occurred at about 18 months of age resulting in death. Pathology showed high levels of virus in the central nervous system, a loss of motor neurons in the spinal cord, and a striking absence of inflammation [45]. Extensive studies subsequently mapped the neurotoxicity to the envelope protein of one such virus, CasBrE [46], but analysis of viral chimeras made with non-neurotropic MuLVs showed that substituted sequences in the viral LTR and *gag-pol* genes significantly reduced the latency period [47]. In these mice, the neurodegeneration is indirectly mediated via infection of glial cells [48]. The effects of this virus on the neurons are independent of the NMDA and AMPA receptors but is dependent on L-type calcium channels [49] and not on cytokines or oxidative stress [46]. Similarly, the envelope proteins of HIV and HERV-K (HML-2) have also been shown to cause neurotoxicity; however the mechanisms seem to be unique to each virus [50].

### Detection of Reverse Transcriptase Activity in ALS:

**Fig. 5:**
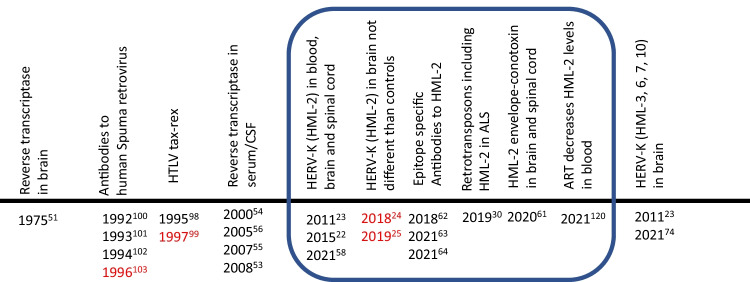
Detection of retroviral elements in ALS. Timeline of detection of retroviral elements in ALS. Years marked in red font indicate where lack of association was reported. Superscripts are the related references. Boxed area shows literature on HML-2 and ALS

The discovery of gamma-retroviruses that could cause paralytic disease in mice and the Visna virus that causes a neurological syndrome in sheep led to the search for similar viruses in individuals with ALS. These retroviruses encode an RNA-dependent DNA polymerase (now termed reverse transcriptase). In 1975, Viola et al. [51] reported high levels of RNA-instructed DNA polymerase/reverse transcriptase activity in extracts from the brains of two individuals with ALS and brain tissue from an asymptomatic patient from the island of Guam but not from control brain samples from the US. The report provides exquisite details of the techniques used. It is notable that the postmortem interval was less than 8 h, and the brain and spinal cord samples were kept and shipped at -70 °C until used. Since Guam had a very high incidence of ALS, it was thought that the study of this population would provide the best chance of identifying an infectious agent. In this report, they also postulated that as in inbred mice studies, the infectious agent maybe produced from an ERV. This was supported by the observation that the Chamorros who immigrate from Guam to the US do not lose their predisposition to developing ALS [52]. They postulated that the “introduction of viral information into the Chamorro germ line could have occurred when the population of Guam was small (latter half of seventeenth century) and its persistence could be the result of a relatively restricted gene pool” [51]. After a hiatus of over a quarter century, following the observation that some individuals with HIV or HTLV-1 infection can develop an ALS-like syndrome, and following the development of the product enhanced reverse transcriptase or PERT assay, several reports emerged demonstrating that reverse transcriptase activity could be found in the blood and CSF of individuals with ALS as well as in smaller numbers of blood relatives or controls [53–56]. However, they were unable to find the source of the reverse transcriptase activity and the search for exogenous retroviruses was unrevealing [53, 54, 57]. While these observations strengthened the possibility of the activation of an ERV in individuals with ALS, the source of the viral activity remained elusive until 2011. Douville et al. made the discovery that the activation of reverse transcriptase gene in brains of ALS subjects arose from HERV-K (HML-2). They demonstrated the presence of both RNA and protein and by immunostaining localized its expression to cortical neurons. Transcripts for the *pol* gene that encodes for reverse transcriptase could also be found in a small number of controls. They cloned and sequenced the transcripts from both ALS individuals and controls and demonstrated that in individuals with ALS there were distinct loci from which the gene was expressed [23]. In a subsequent study, it was shown that all transcripts of HERV-K (HML-2) could be detected in the brain of individuals with ALS and the neurotoxicity was mediated by the expression of the envelope protein (Env) in neurons. Furthermore, transgenic mice that expressed the Env under a neuronal promoter developed an ALS-like syndrome with exclusive degeneration of the upper and lower motor neurons [22]. Other groups have reproduced the discovery of HERV-K activation in individuals with ALS [58, 59] although the activation may not be specific for ALS [24, 25]. HERV-K (HML-2) activation has also been seen in individuals with frontotemporal dementia [58] (Fig. 5).

## Mechanism of HERV-K (HML-2) Induced Motor Neuron Toxicity

There are about 8 HERV-K (HML-2) loci in the human genome that have the capability to express full-length Env. HML-2 Env plays a critical role not only in the packing and infection of viral particles, but in the neurotoxicity and immune activation in the brain. An in vitro study showed that transfection of human neuronal cultures with either *env* construct or whole HML-2 sequence caused significant decrease of both cell numbers and neurite length [22]. Using CRISPR activation technique targeting genomic HML-2 LTR, HML-2 in neurons could be activated. The activation caused neuronal loss and neurite retraction, similar to the effects of *env* plasmid transfection.

A HERV-K (HML-2) *env* transgenic mouse model was generated with neuronal specific Env expression. The expression of Env resulted in neurotoxic effects to the mouse including reduced complexity and length of dendrites, loss of dendritic spines, and beading of the axons and dendrites. Interestingly, the loss of neurons was only observed in the motor cortex. The callosal projection neurons were spared in the transgenic animals. Magnetic resonance imaging showed that only the motor cortex had significant reduction in volume and thickness, consistent with specific motor neuron toxicity induced by the transgene. Behavioral tests showed that the HERV-K (HML-2) *env* transgenic mice had progressive motor function loss with age, recapitulating the symptoms of ALS. By 10 months of age, the transgenic mice had 50% mortality. In summary, this transgenic animal can serve as a good model for motor neuron disease [22].

The surface domain of the HERV-K (HML-2) Env protein was shown to cause neurotoxicity when presented extracellularly. The mechanism involves the interactions with CD98HC and signaling via beta-1 integrin and activation of the GSK-beta pathway. Importantly, this cleaved surface domain of the env protein could be detected in CSF of individuals with ALS and caused neurotoxicity in a similar manner that could be blocked by a monoclonal antibody directed against this epitope [60]. It needs to be determined if the sequence variability of the Env protein from the various loci of HERV-K (HML-2) affects its neurotoxic properties. Recently, it was reported that a novel protein can be translated from the HERV-K *env* transcript through programed ribosomal frameshifting [61]. The protein was termed conotoxin-like protein (CTXLP), which bears a domain with high homology to marine snail conotoxins as well as to HIV Tat protein. CTXLP translation can be induced by pro-inflammatory cytokines. Its expression was associated with motor neuron degeneration, necroptosis, and oligodendrocyte perturbation in ALS. In cell culture models, CTXLP caused apoptotic cell death with caspase-3 cleavage, although there was no caspase activation associated with CTXLP expression in the brain of ALS. Since CTXLP can be translated from the same *env* transcript, it’s important to determine if CTXLP is present in previous experiments with the HERV-K (HML-2) *env* construct. It needs to be determined if CTXLP plays a role in the observed Env neurotoxicity. CTXLP may represent another therapeutic target in ALS.

## Immune Responses to HERV-K (HML-2) in ALS

Specific epitopes of HERV-K (HML-2) Env have been identified as triggering B and T cell responses in individuals with ALS. In an attempt to characterize antibody responses to the HERV-K (HML-2) Env protein it was found that antibody levels to the epitope HERV-K (HML-2) Env-SU _19–37_ significantly correlated with clinical measures of disease severity in individuals with ALS [62]. The antibodies to HERV-K (HML-2) Env protein have a positive correlation with antibodies to TDP-43 [63]. HERV-K Env (HML-2) _19–37_ peptide also induced production of TNF-α in CD8/T cells indicating that this region stimulated both B and T cells. This study also defined another epitope HERV-K (HML-2) Env _109–126_ peptide that activated B cells in vitro [64].

It is worth noting that HERV-K (HML-2) RNA alone has been suggested to function as a ligand for toll-like receptors 7/8 (TLR7/8) in vitro in neurons and microglia, as well as in a mouse model of Alzheimer’s disease, and that TLR activation by HERV-K RNA induces neurotoxicity even in the absence of viral proteins [65]. However, it remains unknown whether endogenously expressed HERV-K transcripts can similarly stimulate innate immune signaling and, if so, whether the interaction is locus-specific.

## Mechanism of HERV-K Activation

The initiating factors that result in activation of HERV-K (HML-2) are largely unknown. The LTR consensus sequence contains a variety of predicted transcription factor binding sites that may affect expression, including NFkB, NFAT, YY1, Sp1/3, androgen and estrogen response elements (ARE/ERE) and interferon-stimulated response elements (ISREs) (reviewed in [66]. Additionally, different HERV-K (HML-2) loci may have fewer putative binding sites or contain unique elements due to insertions or deletions in the promoter. Similarly, HERV-K (HML-2) expression is repressed in healthy tissues by hypermethylation of CpG sites in the LTR, but the level of methylation varies between tissue types and at each specific locus within a given tissue [67–69].

Attempts to activate the viral genome in human neurons in vitro by excitotoxicity, oxidative stress, and histone deacetylation have been unsuccessful [22]. Simultaneous deacetylation and demethylation are necessary for this activation. This is consistent with studies in lymphocytes where histone deacetylase (HDAC) inhibitors alone are not sufficient to reactivate HERV-K, and supports a dual role of histone modifications and DNA methyltransferase in HERV-K repression [70]. This suggests that these retroviral genes are very tightly regulated, likely because their induction can lead to cancer and, as discussed here, neurodegeneration [71]. The transcription factor TDP-43 which is closely involved in the pathophysiology of ALS has five binding sites on the HERV-K (HML-2) LTR [22]. There is a positive correlation between TDP-43 overexpression and HERV-K (HML-2) reactivation in brain tissue from behavioral variant frontotemporal dementia patients [58], which provides additional evidence for a functional role of TDP-43 in HERV-K expression. Another transcription factor, Bcl11b, is a known suppressor of retroviral genes and has been postulated to regulate HERV-K as well. A mutation in this gene has been associated with ALS [72]. Direct nucleosome repositioning that occludes transcription factor binding sites may also be involved; it has been observed that the SMARCB1 component of the SWI/SNF family of chromatin remodeling complexes binds to the LTR and that loss of SMARCB1 upregulates HERV-K (HML-2) expression [21]. Other transcription factors such as FUS that have been implicated in pathophysiology of ALS have not yet been studied in the context of HERV-K activation. Retroviruses can also interact with and regulate gene expression of one another. For example, it has been shown the Tat protein of HIV can regulate expression of HERV-K (HML-2). In blood lymphocytes Tat activates the expression of 26 unique HERV-K (HML-2) proviruses, silences 12, and does not significantly alter expression of the remaining proviruses [73].

## Other Retrotransposons in ALS:

Although most of the transcripts of HERV-K in ALS brain tissue correspond to HML-2, some transcripts have been identified from other subfamilies: HML-3, 6, 7 and 10, and the precise chromosomal loci for these transcripts have been identified [23]. Another group confirmed the expression of HML-6 and localized it to a locus on chromosome 3p21.31c [65]. Network analysis showed co-expression of several chemokine genes and the HIV coreceptors CCR5 and CCR2. It is unknown if the activation of HML-6 at this locus is a consequence of the disease or may play a pathogenic role. However, this locus cannot produce any functional proteins or account for the reverse transcriptase activity in ALS [74]. In a Drosophila model, it has been shown that human TDP-43 expression in glia causes an early and severe dysregulation of the endogenous retrotransposon *gypsy* which leads to neurodegeneration [75]. The TDP-43 homolog in Drosophila, TDPH, modulates siRNA silencing machinery that is responsible for the expression of retrotransposable elements [76]. Gypsy can also be activated by the process of aging and is capable of both cell-associated and cell-free viral transmission which is dependent on the expression of its *env* gene showing virus like properties [77]. TDP-43 has been shown to target a broad range of TEs. This association is lost in frontotemporal dementia and ALS suggesting a dysregulation of TEs [30, 78]. An analysis of transcripts from ALS patient brain tissue revealed that 20% of the samples had elevated expression of TEs. This finding suggests that TE reactivation may represent a molecular subtype of ALS which is distinct from those characterized by increased oxidative stress or predominantly glial cell activation [30].

## Association of Exogenous Retroviruses with ALS-like Syndromes

The discovery of HTLV in the 1970s and the emergence of HIV in the 1980s, along with the ALS-like syndromes observed in subsets of individuals infected with these retroviruses, suggested their direct involvement in the pathogenesis of the disease. These cases sparked renewed interest in the possibility of a retrovirus associated with ALS. Below we review the ALS-like clinical syndromes associated with these two retroviruses.

### Human Immunodeficiency Virus

A constellation of symptoms reminiscent of ALS was first observed in HIV + individuals shortly after the discovery of HIV itself [79, 80]. Since that time there have been numerous cases of an ALS-like syndrome in HIV + individuals reported in the literature [79, 81–84], referred to as HIV-associated ALS (HALS). The individuals described in case reports had a spectrum of ALS-like symptoms and signs noted by the authors, including distal and/or proximal weakness, muscle atrophy, dysarthria, fasciculations in various muscles including the tongue, and hyperreflexia. Electromyography and nerve conduction studies in these individuals frequently found pathology characteristic of ALS, with demonstrable sharp waves, fasciculations, fibrillations, denervation, and absence of sensory findings.

Clinical scoring supported a diagnosis of ALS-like syndrome in many of these cases. Verma and Berger reviewed nineteen cases over a twenty year-period up to 2005. Using El Escorial criteria, they found that four cases met the standard for definite ALS and the rest of the cases met the standard for probable or possible ALS. Satin and Bayat reported three separate cases of HALS with motor limb onset [85]; and Bowen et al. described five cases with motor limb onset, with two of these progressing to involve bulbar symptoms as well [86]. Studies have suggested a prevalence of HALS to be 3.5 cases per 1,000 HIV + individuals [87], in contrast to a prevalence of sporadic ALS (sALS) of one to two cases per 100,000 in the general population [88].

Despite the similarities seen in individuals with HALS and sALS, two comprehensive reviews of HALS cases revealed differences between these two groups [89, 90]. Compared to sALS, individuals with HALS tend to be younger with a mean age of 40 years old while the majority of individuals with sALS were over the age of 55. Their analysis also uncovered a male predilection in HALS, with males affected at a 4.8:1 ratio compared to women, whereas the ratio is ~ 1.5:1 for sALS [91].

The disease course also varies between the two groups, particularly in the setting of combined active antiretroviral therapy (ART). The natural course of sALS almost invariably leads to death approximately 3–5 years from the time of diagnosis. However, in cases of HALS, even though the illness is more severe at onset, trials of antiretroviral therapy have resulted in clinical outcomes ranging from progression to death to complete reversal of symptoms. No consistent relationship has been demonstrated in HALS between HIV viral load or CD4 cell counts in blood to disease progression, response to ART, or final outcomes. However, there is evidence of a temporal relationship between initiation of ART and clinical outcomes, as individuals that started on antiretroviral therapy soon after onset of ALS-like symptoms had better clinical outcomes compared to those with delayed initiation of therapy [86, 90].

### Human T-lymphotropic Virus Type 1

HTLV-1 is a ubiquitous virus, infecting approximately twenty million people worldwide. In the neurological context, it is known to be responsible for HTLV-I associated myelopathy/tropical spastic paraparesis (HAM/TSP). HAM/TSP is a disease that progresses to severe disability (usually wheelchair-bound status) within 20 years of symptom onset [92]. Similar to HIV, HTLV-I has been shown to cause an ALS-like syndrome. Numerous case reports and series [93–97] have described HTLV-1 infected individuals with both upper and lower motor neuron signs including bulbar symptoms and signs, tongue atrophy and fasciculations, limb atrophy, and hyperreflexia.

Nearly forty cases of an ALS-like syndrome in HTLV-1 infected individuals have been reported since the 1980s. The case series by Matsuzaki et al. reported clinical improvement in two of three individuals treated with prednisolone [94]. Ando et al. reported a more recent case of ALS-like syndrome in an HTLV-1 infected patient initially diagnosed with ALS who also responded positively to high-dose steroids. Additionally, they also discuss a case of a patient diagnosed with ALS during life who was found to have clinical features consistent with HAM-TSP on autopsy [96]. While the majority of individuals with HAM-TSP will have additional features not characteristic of ALS (bladder and bowel dysfunction and severe spasticity), the aforementioned cases emphasize the important recognition of an ALS-like syndrome in HTLV-1 infected individuals. Including this phenomenon in the differential diagnosis can allow for the possibility of effective treatment with steroids (and potentially future therapies for HTLV-1) in select individuals. A single report described antibodies to HTLV in patients with ALS and the detection of HTLV tax-rev sequences in blood cells from six ALS patients [98]. These HTLV sequences could not be confirmed by another group [99].

### Spumaviruses

Spuma or Foamy viruses share features of retroviruses and hepadnaviruses. These are non-human viruses but human infections have been described. Antibodies to this virus were described in 25–45% of patients with ALS in a series of publications from a single laboratory [100–102]; however it could not be replicated by another group in which they examined serum and CSF samples from only eight patients with ALS [103].

## Approaches to Targeting Retroviral Elements for Treatment of ALS

### Anti-HERV-K Treatment in Tumor Cell Tines

HERV-K activation and *env* expression were detected in many cancer cell lines. Inhibition of HERV-K in these cells might alter their growth profile. One study found that several pancreatic cancer lines have detectable HERV-K *env* expression [104]. Their culture media showed reverse transcriptase activity and virus-like particles. After knocking down HERV-K using *env*-targeting shRNA, these cells had significantly reduced growth rates in vitro. Also, HERV-K *env* shRNA treatment greatly decreased cancer metastasis to lung in xenograft models. In Atypical Teratoid Rhabdoid Tumor (AT/RT) cells, the deletion or mutation of tumor suppressor gene SMARCB1 may result in elevated HERV-K (HML-2) activation [21]. HERV-K (HML-2) Env protein can be readily detected in these cells. Extracellular vesicles from these cells also contained env protein. Transfection with a HERV-K (HML-2) *env* targeting shRNA down-regulated HERV-K (HML-2) transcription and *env* expression. Cell proliferation was significantly reduced. In some AT/RT cell lines, down-regulation of HERV-K (HML-2) caused cytotoxicity 48 h after shRNA transfection. Similar effects on AT/RT cell growth can be observed by CRISPR interference (CRISPRi) with guide RNA (gRNA) targeting the HERV-K (HML-2) LTR region [21]. It has been proposed that HERV-K (HML-2) Env protein can serve as a tumor associated antigen for cancer treatment. [105–107]. T cells with a chimeric antigen receptor (CAR) specific for HERV-K (HML-2) Env protein (K-CAR) can greatly decrease the proliferation of breast cancer cells and cytotoxicity [108]. In xenograft experiments, K-CAR significantly inhibited tumor growth and metastasis to other organs.

### Anti-HERV-K Antibody Treatment in ALS Cell Culture Models

A HERV-K env specific antibody called GN-K01, developed by GeNeuro also showed preclinical potential as therapy against env toxicity (patent number: US20200308258). CSF from sporadic ALS patient had detectable Env protein. This CSF caused neurite retraction and cell death in cultured human neurons. Treatment with GN-K01 rescued CSF induced neurotoxicity. Similarly, GN-K01 can also block recombinant Env protein induced neuronal death [60]. Thus, neutralizing HERV-K env antibody could potentially be a treatment option for individuals with ALS.

### Anti-retroviral Drugs as Treatment for HERV-K (HML-2)

**Fig. 6:**
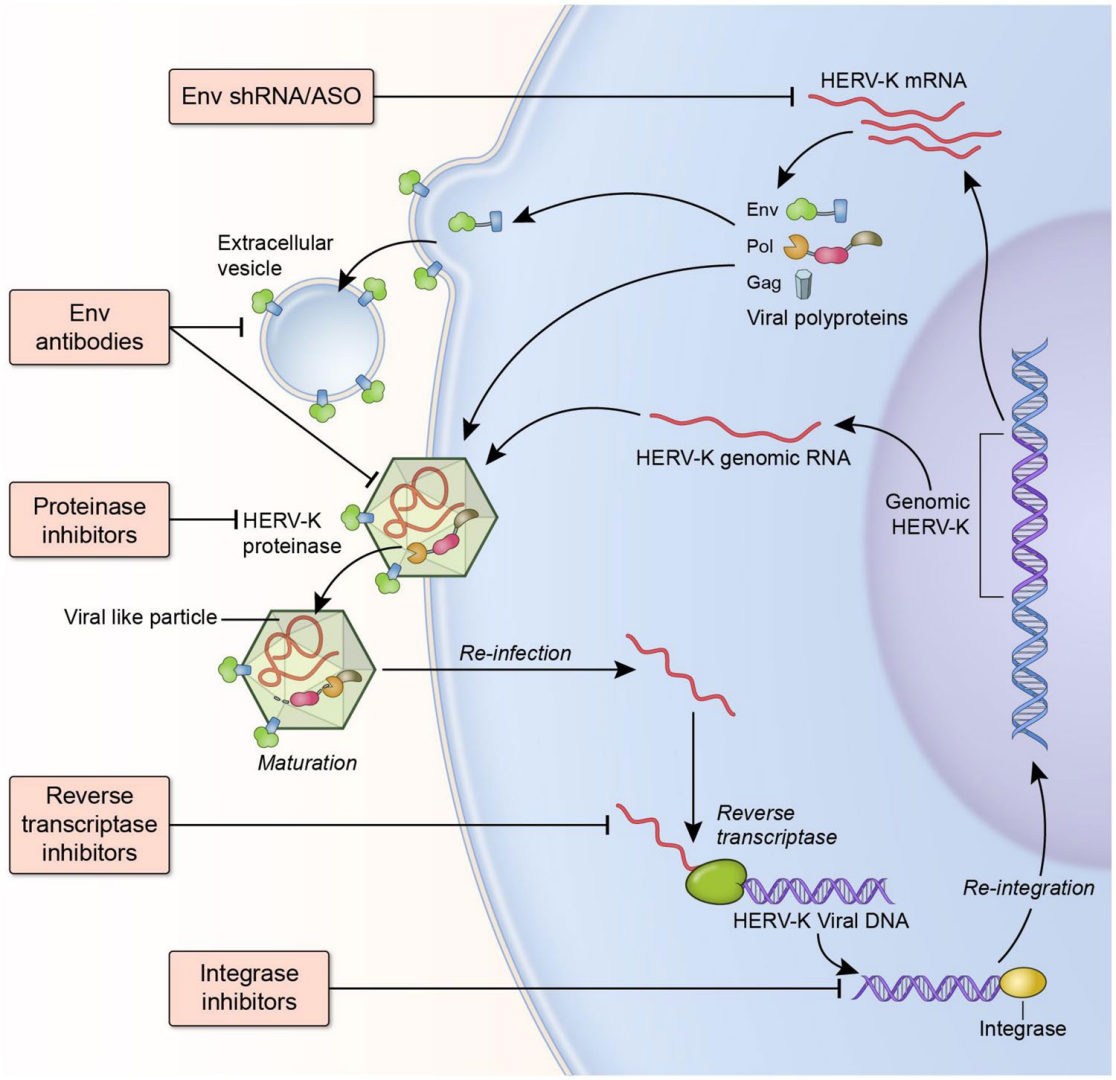
Therapeutic approaches to target HERV-K (HML-2). HERV-K (HML-2) transcripts can be targeted by antisense oligonucleotides (ASO) or shRNA incorporated into adeno-associated viral vectors, extracellular Env can be targeted by monoclonal antibodies, maturation of the viral like particles can be prevented by protease inhibitors, reverse transcription of the viral transcripts to proviral DNA can be prevented by reverse transcriptase inhibitors and incorporation of the proviral DNA into the chromosome can be prevented by integrase inhibitors

Antiretroviral drugs were originally designed to target and treat HIV-1 infection. Currently, there are 32 FDA-approved antiretroviral drugs for HIV/AIDS treatment. They have been hugely effective in suppressing and blocking HIV replication. There are several groups of antiretroviral drugs that specifically target reverse transcriptase, integrase (IN) and proteinase (PRO) (Fig. 6). Early work has shown that nucleotide reverse transcriptase inhibitors have broad activity against several subfamilies of retroviruses [109], although the effective concentration for inhibition varied. Since HERV-K has the same basic genomic structure to HIV, including major genes encoding Env, Gag, and Pol, it could be inhibited by antiretroviral drugs developed for HIV. There are 11 HERV-K (HML-2) proviral genes that can potentially produce the reverse transcriptase protein [110]. Using purified recombinant HERV-K103 RT as enzyme, its activity can be inhibited by some nucleotide reverse transcriptase inhibitors but not by HIV-specific non-nucleotide reverse transcriptase inhibitors [110]. Our group also found that nucleotide reverse transcriptase inhibitors can block HERV-K (HML-2) reverse transcriptase activity, as well as the replication of VSV-G pseudo-typed HERV-K (HML-2) viral particles in a dose-dependent manner [111]. However, we found non-nucleotide reverse transcriptase inhibitors nevirapine, efavirenz, or etravirine can effectively inhibit HERV-K (HML-2) reverse transcriptase activity. Part of the reason might be that we used reverse transcriptase from the consensus HERV-K (HML-2) sequence, while the previous study used HERV-K103 reverse transcriptase. Molecular modeling showed that HIV PRO inhibitors readily dock to the HERV-K (HML-2) PRO catalytic site. Early work reported that HERV-K (HML-2) PRO was resistant to clinically useful HIV proteinase inhibitors ritonavir, indinavir, and saquinavir [112]. It was further found that HERV-K10 PRO can be potently blocked by some proteinase inhibitors belonging to the cyclic urea class [113]. Inhibitors from linear peptidomimetic class were much less effective against HERV-K10 proteinase. In our cell culture model, PRO inhibitor treatment significantly blocked the infectivity of HERV-K (HML-2) viral particles [111]. In addition, IN inhibitor raltegravir was also highly effective against HERV-K (HML-2) replication. Although most antiretroviral drugs were effective against HERV-K (HML-2), the IC90 dosage is higher for HERV-K (HML-2) compared to HIV, especially proteinase inhibitors [110, 114] (see Table 1). So more specific proteinase inhibitors against HERV-K (HML-2) should be developed. In addition, the ability of a drug to enter the brain is an important consideration for potential antiretroviral treatments for ALS. Similar to HIV treatment, more than one drug may be required to completely inhibit HERV-K (HML-2) in patients.

**Table 1:** Comparison of efficacy of antiretroviral drugs against HIV and HERV-K (HML-2)

**Antiretroviral Type**	**Antiretroviral Name**	**HIV IC90 (µM)**	**HIV IC50 (µM)**	**HERV-K (HML-2) IC90 (µM)**	**HERV-K (HML-2) IC50 (µM)**	**Note**
RT inhibitor	Abacavir	2.3		0.175	0.006	tested with consensus HERV-K
	Zidovudine	0.03		0.07	0.008	
	AZTTP	1	0.1		0.55	Tested with HERV-K103
	d4TTP	10	3.16		0.598	
	3TCTP	3.16	0.316		0.809	
	ddCTP	3.16	0.316		0.96	
	ddGTP				0.985	
	ddTTP				0.44	
PRO inhibitor	Darunavir	0.027 to 0.013		0.071	0.029	tested with consensus HERV-K
	Lopinavir		0.017 (no serum) 0.102 ( 50% serum)	0.651	0.034	
IN inhibitor	Raltegravir	0.033		0.075	0.01	

There are several obstacles in achieving optimal antiretroviral drug activity in the brain, which includes low penetration of antiretroviral drugs across the blood brain barrier with reduced tissue concentrations, physiochemical properties of individual antiretroviral drugs (e.g., charge, size, and lipophilicity) that preclude their efficacy in the brain, differential expression and function of transporter proteins on brain cells as well as variable cell type composition, metabolism, and viral replication kinetics (reviewed in [115]. Indeed, quantification of antiretroviral drug concentrations in serum, liver, and different regions of brain tissue, of adult mice at one and four hours after intraperitoneal injection of raltegravir and darunavir showed markedly lower concentrations of both drugs in brain compared to serum and liver [116]. Ferrara and colleagues measured concentrations of antiretroviral drugs in post-mortem brains of 11 HIV-infected persons, receiving different antiretroviral drug regimens and compared these results to published concentrations of antiretroviral drugs in CSF and found that tenofovir, efavirenz and lopinavir had higher concentrations in brain compared to CSF [117]. However, these drugs need to be used with caution since they can cause neurotoxicity [118].

### Monitoring of HML-2 in Patients with ALS

Although HML-2 can be detected in the brain tissue, and biological fluids in research settings there is a need for standardization of assays and for making them widely available. Currently there are no commercially available assays for monitoring HML-2 in biological samples of patients with ALS. Our laboratory is in the process of developing several assays for this purpose. Viral RNA levels are low and unstable but have been detected in CSF [65]. A distinct characteristic of HML-2 is that it can get reverse transcribed to the proviral DNA, within the viral-like particle [119]. Since DNA is stable, it can be easily measured quantitatively by droplet digital PCR in serum, plasma or CSF but needs to be distinguished from chromosomal copies of the viral genes [120]. HML-2 Env protein can also be measured using immunoprecipitation or a sandwich ELISA [59]. Specific epitopes have been identified on the HML-2 Env protein to which ALS patients develop antibodies [62]. Hence antibody levels can also be monitored in these patients. Further standardization of these assays and wide availability would go a long way in monitoring disease progression, determining prognosis and effects of treatment.

### Clinical Studies with Antiretroviral Drugs in ALS

The observation of clinical improvement, and at times resolution, of ALS-like symptoms with antiretroviral drugs in HIV + individuals has prompted clinical trials of anti-retroviral therapy in individuals with sALS with the hope that this class of medication could impact the course of illness. In 2005 a pilot trial with indinavir was conducted in individuals with sALS [121]. Indinavir was chosen due to its anti-apoptotic effect on T lymphocytes and its brain penetrance (which was highest among anti-retroviral medications at that time). Unfortunately, there was no statistical difference in ALS-functional rating scale scores compared to placebo, and the rate of nephrolithiasis was increased on indinavir.

In a study of antiretroviral drugs in HALS, HERV-K (HML-2) levels were measured in conjunction with clinical criteria in three individuals and there was a decrease in HERV-K levels with initiation of anti-retroviral therapy. Activation of HERV-K (HML-2) was localized to Chr 22q11.21 [122]. However, it is unclear if ART directly lowered HERV-K levels, as the HIV Tat protein is known to transactivate HERV-K [73]. The Lighthouse trial was an open-label phase 2a study which confirmed the long-term safety profile of Triumeq (abacavir, lamivudine, and dolutegravir) in individuals with ALS [123], and there is currently a large phase 3 study underway evaluating the efficacy of the medication on overall survival and disease progression in ALS. Analysis of data from the Lighthouse study for levels of HERV-K (HML-2), revealed that treatment with Triumeq correlated with a reduction in HML-2 levels over 24 weeks in eighteen individuals (responders), while four individuals did not show any change from pre-treatment levels (non-responders). From a clinical perspective, while there was no significant difference in ALS-functional rating scale scores between responders and non-responders, non-responders showed a statistically significant decrease in forced vital capacity (FVC) and neurophysiological index (NPI) values compared to responders [120]. Based on these observations, a double-blind placebo-controlled study is now being planned.

### Therapeutic Approaches to Target HERV-K (HML-2) Envelope

Antiretroviral drugs target retroviral enzymes but do not target the Env protein directly. For loci where nearly complete viral sequences are present, this may be a reasonable approach. However, there are additional loci where there is an open reading frame for the envelope but not the other genes. For these loci the antiretroviral drugs would be ineffective, and one would need to directly target these genes. Approaches such as antisense oligonucleotides and shRNA are an excellent strategy for targeting persistent viral infections [124]. Although they can target the transcripts, brain penetration can be a challenge and may require intrathecal delivery and even then, penetration into the brain may be limited. Another possibility is to target the protein with an antibody which might be effective in blocking the protein in the extracellular compartment but not intracellularly (Fig. 6). Hence the best approach might be a combination of antiretroviral drugs and direct targeting of the *env* gene or protein.

In summary, research on the role of retroviral elements in ALS spans over six decades and even though several lines of evidence from that time have pointed to the role of ERVs in the pathophysiology of ALS, the exact nature of the virus remained elusive. Exploration of exogenous infectious agents resulted in multiple dead ends. The discovery of the activation of HERV-K (HML-2) in the pathophysiology of ALS has accelerated the pace of this line of research and early indicators from clinical trials with antiretroviral drugs are encouraging. There is now renewed optimism that targeting these elements may alter the course of this illness in a manner that has not yet been possible by other means.

## Supplementary Information

Below is the link to the electronic supplementary material.

Supplementary file1 (PDF 508 KB)

Supplementary file2 (PDF 517 KB)

Supplementary file3 (PDF 509 KB)

Supplementary file4 (PDF 516 KB)

Supplementary file5 (PDF 508 KB)

Supplementary file6 (PDF 433 KB)
